# Supplementary material for: An exploratory study of associations between judgement bias, demographic and behavioural characteristics, and detection task performance in medical detection dogs
Source: PLoS One. 2025 Apr 9;20(4):e0320158. doi: 10.1371/journal.pone.0320158 (PMC11981131; doi:10.1371/journal.pone.0320158)
Supplement: S1 Table — Including general information, training status when tested and at the data collection endpoint and training outcome. (DOCX) [file pone.0320158.s001.docx]

| **Table S1. MDD sample demographic details.** Including general information, training status when tested and at the data collection endpoint and training outcome. | | | | | | | | | |
| --- | --- | --- | --- | --- | --- | --- | --- | --- | --- |
| **Dog ID** | **Sex** | **Sexual status** | **Dog breed** | **Age in months** | **Task** | **Training**  **stage** | **Status when tested** | **Current fate** | **Training outcome** |
| 1 | Male | Neutered | LR | 19 | Bio | Trainee | Socialiser | Training | Success |
| 2 | Female | Neutered | LG | 17 | Assis | Trainee | Socialiser | Operational | Success |
| 3 | Female | Neutered | LR | 29 | Assis | Trainee | Training | Training | Success |
| 4 | Male | Neutered | LR | 29 | Bio | Trainee | Socialiser | Operational | Success |
| 5 | Male | Neutered | LR | 24 | Bio | Trainee | Socialiser | Failed | Failed |
| 6 | Male | Neutered | LR | 18 | Bio | Trainee | Training | Failed | Failed |
| 7 | Female | Neutered | LG | 25 | Assis | Trainee | Training | Operational | Success |
| 8 | Male | Neutered | LR | 20 | Bio | Trainee | Training | Operational | Success |
| 9 | Female | Neutered | LR | 14 | Bio | Trainee | Socialiser | Operational | Success |
| 10 | Male | Neutered | BC | 113 | Bio | Trained | Retired | Retired | Success |
| 11 | Female | Neutered | LR | 29 | Assis | Trainee | Training | Operational | Success |
| 12 | Male | Neutered | LR | 13 | Bio | Trainee | Socialiser | Training | Success |
| 13 | Female | Neutered | LR | 21 | Bio | Trainee | Socialiser | Training | Success |
| 14 | Female | Neutered | CS | 15 | Bio | Trainee | Training | Failed | Failed |
| 15 | Male | Neutered | LR | 13 | Bio | Trainee | Socialiser | Failed | Failed |
| 16 | Male | Neutered | LR | 15 | Bio | Trainee | Socialiser | Operational | Success |
| 17 | Male | Neutered | LG | 18 | Assis | Trainee | Socialiser | Failed | Failed |
| 18 | Male | Entire | GR | 20 | Bio | Trainee | Socialiser | Operational | Success |
| 19 | Female | Neutered | LR | 17 | Assis | Trainee | Socialiser | Failed | Failed |
| 20 | Male | Neutered | LR | 28 | Bio | Trainee | Socialiser | Traning | Success |
| 21 | Male | Neutered | GR | 20 | Bio | Trainee | Socialiser | Failed | Failed |
| 22 | Female | Entire | LR | 15 | Bio | Trainee | Socialiser | Operational | Success |
| 23 | Female | Entire | CS | 16 | Bio | Trainee | Socialiser | Operational | Success |
| 24 | Female | Neutered | LR | 31 | Assis | Trainee | Socialiser | Operational | Success |
| 25 | Male | Entire | LD | 14 | Assis | Trainee | Socialiser | Operational | Success |
| 26 | Female | Neutered | LR | 54 | Bio | Trained | Operational | Operational | Success |
| 27 | Female | Neutered | LG | 16 | Assis | Trainee | Socialiser | Operational | Success |
| 28 | Female | Neutered | LR | 38 | Bio | Trained | Failed | Failed | Failed |
| 29 | Male | Entire | LG | 14 | Assis | Trainee | Socialiser | Failed | Failed |
| 30 | Male | Neutered | LR | 26 | Bio | Trainee | Socialiser | Failed | Failed |
| 31 | Female | Neutered | LR | 23 | Bio | Trainee | Socialiser | Failed | Failed |
| 32 | Male | Neutered | LG | 14 | Assis | Trainee | Socialiser | Failed | Failed |
| 33 | Female | Neutered | CS | 17 | Bio | Trainee | Training | Failed | Failed |
| 34 | Male | Neutered | LR | 20 | Bio | Trainee | Failed | Failed | Failed |
| 35 | Male | Neutered | LG | 31 | Assis | Trainee | Failed | Failed | Failed |
| 36 | Male | Neutered | LR | 21 | Bio | Trained | Operational | Failed | Failed |
| 37 | Female | Neutered | GR | 26 | Bio | Trained | Operational | Operational | Success |
| 38 | Female | Entire | LR | 13 | Assis | Trainee | Socialiser | Failed | Failed |
| 39 | Male | Neutered | LR | 57 | Bio | Trained | Operational | Operational | Success |
| 40 | Female | Neutered | SS | 52 | Bio | Trained | Operational | Operational | Success |
| 41 | Male | Neutered | CS | 12 | Bio | Trainee | Socialiser | Traning | Success |
| 42 | Female | Neutered | CS | 134 | Bio | Trained | Operational | Retired | Success |
| 43 | Female | Entire | LR | 12 | Assis | Trainee | Socialiser | Operational | Success |
| 44 | Female | Neutered | LG | 81 | Bio | Trained | Operational | Retired | Success |
| 45 | Female | Neutered | LR | 64 | Bio | Trained | Operational | Operational | Success |
| 46 | Female | Neutered | LR | 81 | Bio | Trained | Operational | Retired | Success |
| 47 | Female | Neutered | LR | 36 | Bio | Trained | Operational | Operational | Success |
| 48 | Male | Neutered | CS | 90 | Bio | Trained | Operational | Operational | Success |
| 49 | Male | Neutered | CS | 77 | Bio | Trained | Operational | Operational | Success |
| 50 | Female | Neutered | HV | 104 | Bio | Trained | Operational | Operational | Success |
| 51 | Male | Neutered | LG | 40 | Bio | Trained | Operational | Operational | Success |
| 52 | Male | Neutered | LD | 18 | Assis | Trainee | Training | Operational | Success |
| 53 | Male | Neutered | CS | 20 | Assis | Trainee | Training | Operational | Success |
| 54 | Female | Neutered | LG | 50 | Bio | Trained | Operational | Operational | Success |
| 55 | Male | Neutered | GR | 11 | Bio | Trainee | Socialiser | Operational | Success |
| 56 | Male | Entire | LR | 42 | Bio | Trained | Operational | Training | Success |
| 57 | Female | Entire | LR | 13 | Assis | Trainee | Socialiser | Failed | Failed |
| 58 | Female | Neutered | LR | 35 | Bio | Trained | Operational | Operational | Success |
| *Note.* Bio=Bio-detection dogs, Assis= Assistance dogs, LR= Labrador Retriever, LG= Labrador/Golden cross, GR= Golden Retriever, CS= Cocker Spaniel, SS= Springer Spaniel, LD=Labradoodle, BC=Border Collie, HV= Hungarian wire hair Visla. | | | | | | | | | |
