# Supplementary material for: An exploratory study of associations between judgement bias, demographic and behavioural characteristics, and detection task performance in medical detection dogs
Source: PLoS One. 2025 Apr 9;20(4):e0320158. doi: 10.1371/journal.pone.0320158 (PMC11981131; doi:10.1371/journal.pone.0320158)
Supplement: S2 Table — Subtests description, traits assessed and variables definition. (DOCX) [file pone.0320158.s002.docx]

**S3** **Table. Test battery Parts 1 and 2.** Subtest descriptions, traits assessed, variable definitions, and references to the studies on which the subtests were based.

| **Test battery part 1** | | | |
| --- | --- | --- | --- |
| **Subtest and traits assessed** | **Subtest description** | **Variable** | **Variable Description** |
| S1 Exploring  [1] | This subtest examined the dog's tendency to explore its environment in comparison to an unfamiliar person. The handler entered with the leashed dog and sat near the door, while the tester (SBD) sat opposite, 2 m from the handler. The dog was then unleashed and given two minutes to explore the room. The tester avoided initiating interaction but softly spoke to and petted the dog if it approached. | 1. Duration exploring environment | Time investigating novel room within 1 min (Nose <=10 cm from object, floor or walls) |
| ‘Tendency to investigate humans by sniffing’  ‘Tendency to explore areas by sniffing’  ‘Friendliness to new people’ |  | 2. Duration investigating tester | Time sniffing tester within 1 min (Nose<= 10cm from tester) |
|  |  | 3. Latency to approach tester | Time taken to approach tester within 1 min |
|  |  | 4. Duration interacting with tester | Time making contact or < 50cm, from and paying attention to tester within 1 min. |
|  |  | 5. Duration interacting with handler | Time making contact or <50 cm, from and paying attention to handler within 1 min. |
| S2 Ignoring  [1] | This subtest evaluated the dog's tendency to seek the handler's attention when ignored. The handler and tester sat in the room as in the previous test while the dog roamed freely for three minutes. Throughout the subtest, both the handler and tester ignored the dog. When the subtest concluded, the handler attended to and praised the dog. | 1. Duration interacting with handler | Time making contact or <50cm, from and paying attention to handler within 1 min. |
| ‘Attachment to human partner’ |  | 2. Handler contact frequency | Number of times in contact with their handler within 1 min |
|  |  | 3. Duration interacting with tester | Time making contact or < 50cm, from and paying attention to tester within 1 min. |
|  |  | 4. Tester contact frequency | Number of times in contact with tester within 1 min. |
| S3 Following  [1]  ‘Attachment to human partner’ | This subtest evaluated the dog's inclination to follow its handler as they moved around. The handler was instructed to walk around the room at a normal pace for one minute, ignoring the dog. Then, the handler stopped and attended to the dog. | 1. Duration following handler | Time dog spends making contact or < 50cm, from and paying attention to their handler within 1 min. |
| S4 Reward preference [2] | This subtest examined the dog's preferred reward—either food or toys—and its motivation to obtain each. Two familiarisation trials were conducted in which the tester presented a reward to the dog, held by the handler: one trial with a stuffed toy and the other with a treat in a small bowl. During the testing phase, the dog was presented with both options 3.5m from the dog and the dog was released and allowed to choose and obtain the reward across six pseudorandomised trials. | 1. Frequency choosing food | Number of trials in which the dog chooses and eats food ahead of toy over six trials. |
| ‘Motivation to play with toys’  ‘Motivation to obtain food’ |  | 2. Frequency choosing toy | Number of trials in which the dog chooses and contacts toy ahead of food. Amongst six trials. |
|  |  | 3. Food motivation level | Subjective rating of enthusiasm to reach the chosen reward: 1. Very low to 5. Very high. |
|  |  | 4. Toy motivation level | Subjective rating of enthusiasm to reach the chosen reward: 1. Very low to 5. Very high. |
| S5 Arm pointing  [2-4] | In this subtest, the dog was encouraged to find a hidden treat in a can by following the tester’s pointing gesture. Two cans were placed 3.5 m from the dog, held by the handler, and spaced 1.8 metres apart. Familiarisation involved baiting one can, then both, while the dog watched. The dog was then released and allowed to retrieve treats upon contacting the can. During the test trials, the tester baited one container with a treat, covered the cans with a view blocker, and pointed to the baited can. The dog was then released to choose a can and allowed to take the treat. This was repeated six times in a pseudorandomised order. | 1. Occasions selecting pointed can | Number of trials that the subject's first approach (to <10cm) contact the can or indicate (as in scent discrimination trials) that the tester pointed to within six trials |
| ‘Ability to solve problems’  ‘Independence’ (Cooperation) |  |  |  |
|  |  | 2. Occasions selecting opposite can | Number of trials that the subject's first approach (to <10cm) or contact the can that the tester did not point to within six trials |
| S6 Obedience  [1] | This subtest evaluated the dog's ability to perform three previously learned obedience tasks on cue. The handler stood in the centre of the room facing the dog and was asked to instruct it to 'Sit,' 'Lie down,' and then 'Stay' in sequence, waiting up to 30 seconds before calling the dog. This series of exercises was repeated, and the dog was rewarded with a treat after the second round. | 1. Total latency to obey "Sit" and "Lie down" | Sum of the latencies to perform the "Sit" and "Lie down" tasks each twice |
| ‘Obedience to human command’ |  | 2. Duration Stay | Mean latency before moving after the “Stay” cue is given  maximum 30 sec. |
| S7 Cylinder: Inhibitory control  [2, 5, 6] | This subtest evaluated the dog's ability to restrain a direct response to a visible treat in a transparent hollow cylinder and instead navigate around it to retrieve the treat. During training, the cylinder was covered with dark fabric while the tester introduced the treat, and the dog was allowed to collect it across six trials. In the testing phase, the cover was removed, and the test was repeated six times. The side where the treat was inserted was counterbalanced for each trial. | 1. Immediate detour frequency | Number of times the dog performs a detour to take out the food from one of the cylinder's edges without previously nosing, pushing or pawing the transparent cylinder (out of max six trials) |
| ‘Impulsivity’  ‘Willingness to try new behaviours |  |  |  |
|  |  | 2. Mean latency to obtain food | Average time to extract the food from one of the cylinder's edges over six trials 1 min max. |
|  |  | 3. Different behaviours presented | Total number of different actions presented when trying to access food |
|  |  | 4. Total behaviours presented | Number of behaviours presentedwhen trying to access food |
| S8 Puzzle  [7] | This subtest evaluated the dog’s ability to solve a three-step puzzle and its inclination to either persist independently or seek human assistance. The tester baited the puzzle with 20 treats, placing one in each compartment, and the dog was allowed to extract them for three minutes. | 1. Number of treats recovered | Number of food items located and consumed (out of 20) within 3 minutes |
| ‘Ability to solve problems’  ‘Independence’  ‘Persistence’ |  | 2. Duration on task | Time engaged in locating food over 3 minutes (sec) |
|  |  | 3. Latency to gaze at human | Time before looking at tester or handler within 3 minutes (sec) |
|  |  | 4. Duration gazing human | Total time (within 3 min) looking at tester or handler |
|  |  | 5. Duration not on task | Total time (within 3 min) not focused on finding treats |

| **Test battery part 2** | | | |
| --- | --- | --- | --- |
| **Subtest and traits assessed** | **Subtest description** | **Variable** | **Variable Description** |
| S9 Boxes search  [1, 8] | This subtest examined the dogs' ability to use their sense of smell to locate hidden food. Four transparent containers filled with paper shreds were arranged in a semi-circle, 25 cm apart, in the centre of the room. The tester baited one container with a treat and pretended to bait the others to prevent the dog from visually identifying the baited box. The dog was then released to search for the treat within one minute. If successful, the handler praised the dog and the trial ended; this process was repeated three times. | 1. Latency to find treats | Mean latency to find food item over three trials (1 min max each) |
| ‘Acuity of sense of smell’  ‘Tendency to remain specific to the target odour’ |  | 2. Number of treats located | Total food items found and consumed over three trials (maximum 3) |
|  |  | 3. Time not on task | Mean percentage of the total time not focused on finding treats over three trials (1 min max each) |
| S10 Noise distraction  [8, 9]  ‘Tendency to be distracted when working’ | This subtest evaluated the dogs’ ability to focus on a search task despite distracting noise. The previous search task (S9) was repeated, but a recording of an unfamiliar sound (a Geiger counter) was played during the one-minute search. The sound was played three times at random intervals throughout the trial. | 1. Time attending to noise | Duration oriented and apparently focussed on the sound (max 30 sec) |
| S11 Unsolvable task  [2, 10] | This subtest evaluated the dog’s tendency to persist independently in retrieving an out-of-reach treat or to seek human assistance. For familiarisation, the tester baited a transparent container with a high-value treat, allowing the dog to sniff it. Initially, the container was placed in the room with a partially open lid, enabling the dog to access the treat within one minute. For testing, the procedure was repeated with the container fully closed, making retrieval impossible, and the dog was allowed one minute to attempt to access the treat. | 1. Duration on task | Time attempting to access food items (1 min max) |
| ‘Ability to solve problems’  ‘Independence’  ‘Persistence’  ‘Willingness to try new behaviours’ |  | 2. Duration gazing at human | Total duration dog looked towards the tester or handler over 1 min. |
|  |  | 3. Duration gazing at tester | Time dog looked towards the tester over 1 min. |
|  |  | 4. Duration gazing at handler | Time dog looked towards handler over 1 min. |
|  |  | 5. Latency to gaze at tester | Time before dog looks towards tester maximum 1 min. |
|  |  | 6. Latency to gaze at handler | Time before dog looks towards handler within 1 min. |
|  |  | 7. Latency to gaze at human | Latency to gaze human within 1 min. |
|  |  | 8. Activity alternation frequency | Number of changes from task to other activity and vice-versa |
|  |  | 9. Number of different behaviours presented | Different behaviours presented during task |
| S12 Ball searching  Based on a test from Medical Detection Dogs® [11] | This subtest assessed a dog’s ability to locate a hidden ball using its sense of smell. The tester placed three upside-down metal cans in a row, spaced 25 cm apart. The handler sat on the floor, holding the dog 50 cm away from the cans. Initially, the tester threw a tennis ball three times to encourage the dog to retrieve it.  During the testing trials, the tester hid the ball under the target can and then shuffled it according to a set pattern to make the dog lose sight of the target can and rely on its sense of smell. The dog was then released to find the ball, receiving praise and a retrieval throw if successful; if not, the handler encouraged further searching. | 1. Total correct choices | Number of choices of the baited pot (first container they contact with snout or paw or indicate with trained alert 0= fail, 1= found with assistance from tester 2= found (scoring 0-6) |
| ‘Acuity of sense of smell’  ‘Tendency to remain specific to the target odour’  ‘Willingness to bring an object back to a person‘ |  |  |  |
|  |  | 2. Ball retrieval score | Number of occasions when the ball is retrieved to the tester  0= not retrieved, 1= retrieved with intervention (speaking to dog or slightly puling ball from mouth), 2= retrieved to handler or tester (scoring 0-12) |
| S13 Toy playing  [1] | This subtest evaluated the dog’s inclination to engage in social play with a human using a toy. The handler encouraged the dog to play tug-of-war with a familiar stuffed toy for one minute. Afterwards, the handler instructed the dog to release the toy and perform a brief obedience sequence—sit, lie down, and stay for 30 seconds—similar to the sequence in S6, but only once. | 1. Duration playing | Time interacting with toy over 60 seconds |
| ‘Motivation to play with toys’  ‘General excitability’ |  | 2. Activity alternation frequency | Number of changes from playing with toy to other activity and vice-versa |
|  |  | 3. Play intensity | Subjective rating for involvement with tug of war game on a scale of 1 - very low to 5 - very high |
|  |  | 4. Variety of behaviours presented | Number of different behaviour types presented when playing |
|  |  | 5. Latency to drop | Mean time to drop toy after being instructed by the handler  maximum 1 min |
|  |  | 6. Total latency to obey "Sit" and "Lie down" | Sum of the latencies to perform the "Sit" and "Lie down" tasks once |
|  |  | 7. Duration Stay | Latency before moving after the “Stay” cue is given  maximum 30 sec. |
| S14 Coat wearing | This subtest assessed the dog's tolerance for wearing a service coat. The handler dressed the dog in the coat in the centre of the room and returned to their seat. The dog was allowed to move freely around the room for 30 seconds, with the handler interacting normally if approached but without intentionally encouraging excitement. | 1. Level of restraint required | Subjective rating for the level of restraint required when putting coat on 1 very low- Staying still, relaxed o 5 very high-Does not tolerate |
| ‘Body sensitivity’ |  |  |  |
|  |  | 2. Biting coat frequency | Number of times dog places coat in their mouth within 30 sec |
|  |  | 3. Frequency of approaches to human | The dog makes contact or < 50cm from handler or tester |
| S15 Body condition check-up  [12] | This subtest evaluated the dog’s tolerance for a body condition check-up. The handler held the dog in the centre of the room while the tester performed a basic body examination, similar to a veterinarian's assessment, handling the dog around the head and body while providing treats. The tester also used a stethoscope to examine the dog. | 1. Level of tolerance of check-up | Subjective rating for the level of dog’s acceptance of a veterinary check-up with a scale of 1 to 5: 1 very low - Tries to escape to 5 very high - Stays still, relaxed |
| ‘Body sensitivity’  ‘Confidence’ |  |  |  |
| S16 Novel object  [1] | This subtest assessed the dog's tendency to interact with or avoid an unfamiliar moving object. The tester took out a motorised toy that jumps and makes noise (Bumble Ball®) from a fabric bag, activated it, and placed it 1.5 metres away from the dog, which was held by the handler. The handler then released the dog to explore the toy freely without any encouragement. After approximately 20 seconds, when the toy stopped moving, the tester retrieved it and put it back in the bag. | 1. Level of approach towards new object | Subjective ratings of dog's response to moving ball object: 1 Very low/retreat to 5 to Very high/Approaches to object |
| ‘Confidence’ |  | 2. Number of different behaviours towards new object | Total variety of behaviours displayed when the dog is presented with the moving object |
| S17 Slippery surface  [1] | This subtest evaluated the dog's willingness to step onto an unfamiliar surface to reach food rewards. A sheet of polished wood was placed alongside a smooth acrylic surface, creating a larger area for the dogs to walk over and enhancing the novelty with two different materials. The handler holding the dog positioned themselves near the surface while the tester scattered treats across it. The dog was then released to explore the surface and collect the treats. | 1. Latency to step onto surface | Time taken to place all four paws on the slippery surface maximum 30 sec. |
| ‘Confidence in different environments’ |  | 2. Duration on slippery surface | Total time with four paws n on novel surface maximum 30 sec. |
|  |  | 3. Readiness of approach to slippery surface | Subjective rating of dog’s response to a novel surface. Scale: 1 Very low-does not step on surface to 5 - Readily -jumps straight onto surface |
| S18 Holding pen  [1] | This subtest assessed the dogs' ability to settle without human presence. Once the main test concluded, the handler placed the dog in a crate, pen, room, or holding area where they were typically left. The dog was then filmed with a GoPro camera installed in the room for five minutes without any human companionship. After this time, some dogs were removed, while others remained, based on the handler's decision. | 1. Latency to rest | Latency to lie down |
| ‘Ease of adaptation to crate or kennel‘ |  | 2. Duration resting | Total time lying down |
|  |  | 3. Duration moving | Total time walking in pen within 5 minutes |
|  |  | 4. Duration exploring | Total time investigating environment within 1 min (nose <10cm from object, floor or walls/ fences) |
|  |  | 5. Duration standing | Total time standing in pen within 5 minutes |
|  |  | 6. Pen gate approach frequency | Number of times the dog approaches from within 20cm or less of the holding pen gate within 5 minutes |
|  |  | 7. Pawing on pen or enclosure area | Number of times the dog places their paw on the gate or walls/ fences within 5 minutes |
|  |  | 8. Jumping up frequency | Number of times when the dog jumps or climbs up supported on its back paws within 5 minutes |
|  |  | 9. Duration vocalising | Total time vocalising whilst in holding in pen within 5 minutes |
|  |  | 10. Vocalisation frequency | Total number of vocalisations made whilst in holding pen within 5 minutes |

**References**

1. Rooney NJ, Bradshaw JWS, Gaines SA. UK Detection Dog Rearing Project - an investigation into factors affecting search ability. 2003.

2. MacLean EL, Herrmann E, Suchindran S, Hare B. Individual differences in cooperative communicative skills are more similar between dogs and humans than chimpanzees. Anim Behav. 2017;126:41-51.

3. Hare B, Call J, Tomasello M. Communication of food location between human and dog (Canis familiaris). Evol Commun. 1998;2(1):137-59.

4. Miklösi Á, Polgárdi R, Topál J, Csányi V. Use of experimenter-given cues in dogs. Anim Cogn. 1998;1(2):113-21.

5. Bray EE, MacLean EL, Hare BA. Context specificity of inhibitory control in dogs. Anim Cogn. 2014;17(1):15-31.

6. Tiira K, Tikkanen A, Vainio O. Inhibitory control–Important trait for explosive detection performance in police dogs? Appl Anim Behav Sci. 2020;224:104942.

7. Bray EE, Sammel MD, Seyfarth RM, Serpell JA, Cheney DL. Temperament and problem solving in a population of adolescent guide dogs. Anim Cogn. 2017;20(5):923-39.

8. Svartberg K. Shyness-boldness predicts performance in working dogs. Appl Anim Behav Sci. 2002;79(2):157-74.

9. Batt LS, Batt MS, Baguley JA, McGreevy PD. Factors associated with success in guide dog training. J Vet Behav. 2008;3(4):143-51.

10. Miklosi A, Kubinyi E, Topal J, Gacsi M, Viranyi Z, Csanyi V. A simple reason for a big difference: Wolves do not look back at humans, but dogs do. Curr Biol. 2003;13(9):763-6.

11. Medical Detection Dogs. About us. 2020. Retrieved from https://www.medicaldetectiondogs.org.uk/about-us/ 2020 [cited 2021 05/06/2021]. Available from: <https://www.medicaldetectiondogs.org.uk/about-us/>.

12. Harvey ND, Craigon PJ, Sommerville R, McMillan C, Green M, England GCW, et al. Test-retest reliability and predictive validity of a juvenile guide dog behavior test. J Vet Behav. 2016;11:65-76.
