# Supplementary material for: An exploratory study of associations between judgement bias, demographic and behavioural characteristics, and detection task performance in medical detection dogs
Source: PLoS One. 2025 Apr 9;20(4):e0320158. doi: 10.1371/journal.pone.0320158 (PMC11981131; doi:10.1371/journal.pone.0320158)
Supplement: S1 Appendix — Table A shows the ranges of scent sensitivity and specificity across conditions. Table B includes the number of exposures and presented scent samples, target source, and the number of training and testing trials for each target scent. (DOCX) [file pone.0320158.s003.docx]

**S2 Appendix. Additional information on scent sensitivity and specificity conditions and scent exposures.**

**Table A.** Ranges of sensitivity and specificity scores for dogs trained on each target scent (N= number of dogs = 27).

| Target scent | N | Sensitivity | | | Specificity | | |
| --- | --- | --- | --- | --- | --- | --- | --- |
|  |  | Mean ±SD | Min | Max | Mean ± SD | Min | Max |
| Covid-19 | 13 | 0.80 ±0.12 | 0.51 | 1.00 | 0.89 ±0.11 | 0.70 | 1.00 |
| Pseudomonas | 3 | 0.67 ±0.21 | 0.50 | 0.90 | 0.92 ±0.034 | 0.88 | 0.94 |
| Parkinson's | 2 | 0.85 ±0.21 | 0.70 | 1.00 | 0.89 ±0.014 | 0.88 | 0.90 |
| Malaria | 1 | 0.80 ±0.00 | 0.80 | 0.80 | 0.92 ±0.00 | 0.92 | 0.92 |
| Prostate Cancer | 4 | 0.80 ±0.11 | 0.71 | 0.92 | 0.82 ±0.12 | 0.70 | 0.98 |
| Canine Bladder Cancer | 2 | 0.85 ±0.14 | 0.84 | 0.86 | 0.89 ±0.08 | 0.84 | 0.95 |
| Amyl Acetate (canine olfactory threshold trial) | 2 | 0.78 ±0.30 | 0.57 | 0.99 | 0.91 ±0.01 | 0.90 | 0.91 |
| All | 27 | 0.79 ±0.14 | 0.50 | 1.00 | 0.88 ±0.09 | 0.70 | 1.00 |

**Table B.** Number of exposures and presented samples, target source, and number of training and testing trials for each target scent (NB. Medical Detection Dogs were unable to provide complete information for some conditions). (N= number of dogs = 27).

| Target scent | N | Exposures nº | | | Samples nº | | | Material/ scent | Trial (N) | | |
| --- | --- | --- | --- | --- | --- | --- | --- | --- | --- | --- | --- |
|  |  |  |  |  |  |  |  |  | *Training* | | *Testing* |
|  |  | Mean ± SD | Min | Max | Mean ± SD | Min | Max |  | Unblinded | *blinded* |  |
| Covid-19 | 13 | 1069.92 ±1033.22 | 14 | 2811 | 201.77 ±196.89 | 5 | 425 | Sock- Tennis Ball | 6 | 2 | 5 |
| Pseudomonas | 3 | - | - | - | 104± | - | - | Pa in mixed culture with other organisms | 0 | 0 | 3 |
| Parkinson's | 2 | - | - | - | - | - | - | - | 0 | 0 | 2 |
| Malaria | 1 | 3125 | 3125 | 3125 | 340 ±0 | 340 | 340 | Sock | 0 | 1 | 0 |
| Prostate Cancer | 4 | - | - | - | - | - | - | Urine | 2 | 0 | 2 |
| Canine Bladder Cancer | 2 | 790 ±41.01 | 761 | 819 | 212 ±2.12 | 211 | 214 | Urine | 0 | 0 | 2 |
| Amyl acetate | 2 | 870 ±182.43 | 741 | 999 | 29 ±11.31 | 21 | 37 | Amyl Acetate solution | 0 | 1 | 1 |
| All | 27 | 1130.78 ±1007 | 14 | 3125 | 186.84 ±174.75 | 5 | 425 | - | 8 | 4 | 15 |
|  |  |  |  |  |  |  |  | Total of each trial sort % | 29.6% | 14.8% | 55.6% |

*Note.* For 15 dogs, sensitivity and specificity were calculated from testing trials across conditions (except Malaria, where testing data was unavailable). For Prostate Cancer, Pa [41] and Parkinson's disease, data was from testing trials only. Some dogs trained on COVID-19, Canine Bladder Cancer, Malaria, and Canine olfactory threshold trial (COT) [42] did not yet have testing trials or were withdrawn before testing. Therefore, We used the most recent and advanced level of training data. The COT project data was from an experiment investigating the dogs' olfactory threshold for biodetection purposes with amyl acetate, a compound frequently utilised for olfactory studies.
