# Supplementary material for: An exploratory study of associations between judgement bias, demographic and behavioural characteristics, and detection task performance in medical detection dogs
Source: PLoS One. 2025 Apr 9;20(4):e0320158. doi: 10.1371/journal.pone.0320158 (PMC11981131; doi:10.1371/journal.pone.0320158)
Supplement: Attachment — Submitted filename: Response to Reviewers Bistre et al.docx [file pone.0320158.s005.docx]

*We are grateful for your review and the insightful comments and suggestions you provided. We are confident that the paper has improved during the revision process, and we hope you share this view. Our responses are presented in italics below. We included line numbers in our responses to indicate the suggested changes made in the manuscript.*

Reviewers' comments:

Reviewer's Responses to Questions

**Comments to the Author**

1. Is the manuscript technically sound, and do the data support the conclusions?

The manuscript must describe a technically sound piece of scientific research with data that supports the conclusions. Experiments must have been conducted rigorously, with appropriate controls, replication, and sample sizes. The conclusions must be drawn appropriately based on the data presented.

Reviewer #1: Yes

2. Has the statistical analysis been performed appropriately and rigorously?

Reviewer #1: Yes

3. Have the authors made all data underlying the findings in their manuscript fully available?

The [PLOS Data policy](https://gbr01.safelinks.protection.outlook.com/?url=http%3A%2F%2Ftrack.editorialmanager.com%2FCL0%2Fhttp%3A%252F%252Fwww.plosone.org%252Fstatic%252Fpolicies.action%2523sharing%2F1%2F010f0193dc92e79f-26641ac6-a085-4062-bf0d-b516bc837041-000000%2FJrMq_LEO5xEWzHicFzMuH8izS_1AW7t2wO__4lQSqtI%3D190&data=05%7C02%7C%7Cdf03dd01eeff47251de708dd210ceba5%7C84df9e7fe9f640afb435aaaaaaaaaaaa%7C1%7C0%7C638703061563719673%7CUnknown%7CTWFpbGZsb3d8eyJFbXB0eU1hcGkiOnRydWUsIlYiOiIwLjAuMDAwMCIsIlAiOiJXaW4zMiIsIkFOIjoiTWFpbCIsIldUIjoyfQ%3D%3D%7C0%7C%7C%7C&sdata=ua9AeVLFAnJwamklAjwBo8J0tGcx%2BEfIS2GXJn9WTGg%3D&reserved=0) requires authors to make all data underlying the findings described in their manuscript fully available without restriction, with rare exception (please refer to the Data Availability Statement in the manuscript PDF file). The data should be provided as part of the manuscript or its supporting information, or deposited to a public repository. For example, in addition to summary statistics, the data points behind means, medians and variance measures should be available. If there are restrictions on publicly sharing data—e.g. participant privacy or use of data from a third party—those must be specified.

Reviewer #1: Yes

4. Is the manuscript presented in an intelligible fashion and written in standard English?

PLOS ONE does not copyedit accepted manuscripts, so the language in submitted articles must be clear, correct, and unambiguous. Any typographical or grammatical errors should be corrected at revision, so please note any specific errors here.

Reviewer #1: Yes

5. Review Comments to the Author

Please use the space provided to explain your answers to the questions above. You may also include additional comments for the author, including concerns about dual publication, research ethics, or publication ethics. (Please upload your review as an attachment if it exceeds 20,000 characters)

Reviewer #1: The manuscript is well-written and organized, following a clear roadmap of events. The introduction provides a compelling rationale for the study, and the methods are described with sufficient detail, allowing for reproducibility.

The statistical analyses are robust and thoroughly explained, and the results are presented in a logical sequence that mirrors the research questions, making it easy to follow the progression of the study.

This is a very interesting and innovative piece of work that explores the relationship between personality traits and decision-making in medical detection dogs. The findings provide valuable insights into how affective and cognitive processes might influence detection performance. By identifying traits such as optimism, confidence, and behavioral tendencies that correlate with task outcomes, the study opens the door to further research in this area.

Additionally, the study highlights the potential utility of judgment bias tasks as predictive tools, which could have implications for the selection and training of other types of detection dogs. The work has significant practical and theoretical implications, making it a strong contribution to the field.

I have no major concerns, but I would encourage the authors to consider:

The authors should consider discussing alternative explanations for why the middle position was significantly selected by the dogs. While the findings suggest that more playful and food-motivated dogs approached the middle position faster, interpreted as optimism, the association between higher specificity and slower approaches to the same position suggests a more cautious, discriminative behavior. This raises questions about the underlying factors influencing middle position selection. For instance, could the middle position's significance be related to training protocols, novelty of the setup, or the fact that the tester was positioned behind this area? It is possible that dogs heard subtle cues from the tester or other background noise, leading them to anticipate something about the task. Exploring whether this position inherently holds some predictive or environmental cues could provide deeper insights into the observed behaviors and refine interpretations of optimism versus discrimination tendencies.

Overall, this is an interesting study with the potential to inspire further research in both medical and other detection dog applications.

*Thank you for this interesting comment. In our view, and as outlined in lines 610-614 of the original paper, the most plausible reason for significant differences being detected at the middle rather than the other ambiguous cue locations is that, due to its central location, it presented the highest level of ambiguity to the dogs. As such, responses to it are most likely to have been ‘vulnerable’ to influence by individual-specific states and traits, and hence to reveal associations with related predictor variables. It is perhaps also possible that ambiguity of the middle position was further increased by noise or other cues from the tester positioned behind this location despite measures taken to minimise such effects, including visual occlusion. Any such unintentional cues are likely to have varied between trials and hence not to have exerted a consistent inflence on responses. We have edited the text to include these points (Line 610-619 Revised Manuscript Clean).*

6. PLOS authors have the option to publish the peer review history of their article ([what does this mean?](https://gbr01.safelinks.protection.outlook.com/?url=https%3A%2F%2Ftrack.editorialmanager.com%2FCL0%2Fhttps%3A%252F%252Fjournals.plos.org%252Fplosone%252Fs%252Feditorial-and-peer-review-process%2523loc-peer-review-history%2F1%2F010f0193dc92e79f-26641ac6-a085-4062-bf0d-b516bc837041-000000%2FuCo0LQMu0Yl2VxSD-PnJilrWAdTe3K1oG2MyuOjrxIQ%3D190&data=05%7C02%7C%7Cdf03dd01eeff47251de708dd210ceba5%7C84df9e7fe9f640afb435aaaaaaaaaaaa%7C1%7C0%7C638703061563733467%7CUnknown%7CTWFpbGZsb3d8eyJFbXB0eU1hcGkiOnRydWUsIlYiOiIwLjAuMDAwMCIsIlAiOiJXaW4zMiIsIkFOIjoiTWFpbCIsIldUIjoyfQ%3D%3D%7C0%7C%7C%7C&sdata=TYPHmNQC57G1QVZ%2B0hseteo33X60sEcjQC%2FfY%2F7Ldas%3D&reserved=0)). If published, this will include your full peer review and any attached files.


If you choose "no", your identity will remain anonymous but your review may still be made public.


**Do you want your identity to be public for this peer review?** For information about this choice, including consent withdrawal, please see our [Privacy Policy](https://gbr01.safelinks.protection.outlook.com/?url=https%3A%2F%2Ftrack.editorialmanager.com%2FCL0%2Fhttps%3A%252F%252Fwww.plos.org%252Fprivacy-policy%2F1%2F010f0193dc92e79f-26641ac6-a085-4062-bf0d-b516bc837041-000000%2FBMcFw0pS6jCGkG0LdJYpvz1uWBmLKJhhSyxtVCJ1lTM%3D190&data=05%7C02%7C%7Cdf03dd01eeff47251de708dd210ceba5%7C84df9e7fe9f640afb435aaaaaaaaaaaa%7C1%7C0%7C638703061563747234%7CUnknown%7CTWFpbGZsb3d8eyJFbXB0eU1hcGkiOnRydWUsIlYiOiIwLjAuMDAwMCIsIlAiOiJXaW4zMiIsIkFOIjoiTWFpbCIsIldUIjoyfQ%3D%3D%7C0%7C%7C%7C&sdata=cQDNqeLqLXjVMSxmrUH9guoA3jr7V4PS9km0rSwZmqc%3D&reserved=0).

Reviewer #1: **Yes:** Astrid R. Concha
